# Supplementary material for: What do adolescents with asthma really think about adherence to inhalers? Insights from a qualitative analysis of a UK online forum
Source: BMJ Open. 2017 Jun 13;7(6):e015245. doi: 10.1136/bmjopen-2016-015245 (PMC5734261; doi:10.1136/bmjopen-2016-015245)
Supplement: Supplementary data [file bmjopen-2016-015245supp002.docx]

**COREQ checklist (Tong, Sainsbury and Craig, 2007)**

*What do adolescents with asthma really think about adherence to inhalers? Insights from a qualitative analysis of a UK online forum*

**Domain 1: Research team and reflexivity**

*Personal Characteristics*

1. Interviewer/facilitator

Which author/s conducted the interview or focus group? N/A

2. Credentials

What were the researcher’s credentials? E.g. PhD, MD First author: PhD Last Author: PhD

3. Occupation

What was their occupation at the time of the study? First author: NIHR Academic Clinical Lecturer in Primary Care Research

4. Gender

Was the researcher male or female? Female

5. Experience and training

What experience or training did the researcher have? First author: Qualitative research training embedded into Academic Clinical Fellowship in Primary Care Research, University of Cambridge. Experience in qualitative research:

- De Simoni A, Shanks A, Balasooriya-Smeekens C, Mant J. Stroke survivors and their families receive information and support on an individual basis from an online forum: descriptive analysis of a population of 2348 patients and qualitative study of a sample of participants. BMJ Open 2016;6:e010501.doi: 10.1136/bmjopen-2015 010501
- Balasooriya-Smeekens C, Bateman A, Mant J, De Simoni A. Barriers and facilitators to staying in work after stroke: insight from an online forum. BMJ Open 2016;6:e009974. doi: 10.1136/bmjopen-2015-009974.
- Hardeman W, Lamming L, Kellar I, De Simoni A, Boase S, Graffy J, Boase S, Sutton S, Farmer A, Kinmonth AL. Implementation of a nurse-led behaviour change intervention to support medication taking in type 2 diabetes: beyond hypothesised active ingredients (SAMS Consultation Study). (2014) Implement Sci. 2014 5;9:70. doi: 10.1186/1748-5908-9-70.
- De Simoni A, Shanks A, Mant J, Skelton J. Making sense of patients’ internet forums: a systematic method using discourse analysis. (2014) Br J Gen Pract. 64 (620), e178-e180.

*Relationship with participants*

6. Relationship established

Was a relationship established prior to study commencement? N/A

7. Participant knowledge of the interviewer

What did the participants know about the researcher? e.g. personal goals, reasons for doing the

research N/A

8. Interviewer characteristics

What characteristics were reported about the interviewer/facilitator? e.g. Bias, assumptions, reasons

and interests in the research topic N/A

**Domain 2: study design**

*Theoretical framework*

9. Methodological orientation and Theory

What methodological orientation was stated to underpin the study? e.g. grounded theory, discourse

analysis, ethnography, phenomenology, content analysis. Thematic analysis, p 8.

*Participant selection*

10. Sampling

How were participants selected? e.g. purposive, convenience, consecutive, snowball. Purposive, p. 7-8

11. Method of approach

How were participants approached? e.g. face-to-face, telephone, mail, email N/A

12. Sample size

How many participants were in the study? 54, p. 10.

13. Non-participation

How many people refused to participate or dropped out? Reasons? N/A

*Setting*

14. Setting of data collection

Where was the data collected? e.g. home, clinic, workplace Online forum, p. 6-9

15. Presence of non-participants

Was anyone else present besides the participants and researchers? N/A

**Page 26 of 27**

**For peer review only - http://bmjopen.bmj.com/site/about/guidelines.xhtml**

16. Description of sample

What are the important characteristics of the sample? e.g. demographic data, date Adolescents with asthma discussing inhaler treatment. Parents of adolescents with asthma and adults with asthma who were registered users of the online forum and discussing with adolescents issues with taking inhalers. p.10.

*Data collection*

17. Interview guide

Were questions, prompts, guides provided by the authors? Was it pilot tested? N/A

18. Repeat interviews

Were repeat interviews carried out? If yes, how many? N/A

19. Audio/visual recording

Did the research use audio or visual recording to collect the data? N/A

20. Field notes

Were field notes made during and/or after the interview or focus group? N/A

21. Duration

What was the duration of the interviews or focus group? N/A

22. Data saturation

Was data saturation discussed? Yes, p.8.

23. Transcripts returned

Were transcripts returned to participants for comment and/or correction? N/A

**Domain 3: analysis and findings**

Data analysis

24. Number of data coders

How many data coders coded the data? 2

25. Description of the coding tree

Did authors provide a description of the coding tree? Table 2 at p. 31 reports all themes and codes was included.

26. Derivation of themes

Were themes identified in advance or derived from the data? Derived from the data (however

purposively looking for barriers and facilitators), p.8-9.

27. Software

What software, if applicable, was used to manage the data? Microsoft Excel, NVivo11, p.9.

28. Participant checking

Did participants provide feedback on the findings? N/A

*Reporting*

29. Quotations presented

Were participant quotations presented to illustrate the themes/findings? Yes, however, for ethical

reasons quotes were presented as descriptions rather than quotes literally extracted

from the online forum.p. 6-7.

Was each quotation identified? e.g. participant number Yes, p. 10-22.

30. Data and findings consistent

Was there consistency between the data presented and the findings? Yes, p. 10-23.

31. Clarity of major themes

Were major themes clearly presented in the findings? Yes, p. 10-23, table 2 p.30.

32. Clarity of minor themes

Is there a description of diverse cases or discussion of minor themes? Yes, p. 11-23.
