# Supplementary material for: What do adolescents with asthma really think about adherence to inhalers? Insights from a qualitative analysis of a UK online forum
Source: BMJ Open. 2017 Jun 13;7(6):e015245. doi: 10.1136/bmjopen-2016-015245 (PMC5734261; doi:10.1136/bmjopen-2016-015245)
Supplement: Supplementary data [file bmjopen-2016-015245supp001.pdf]

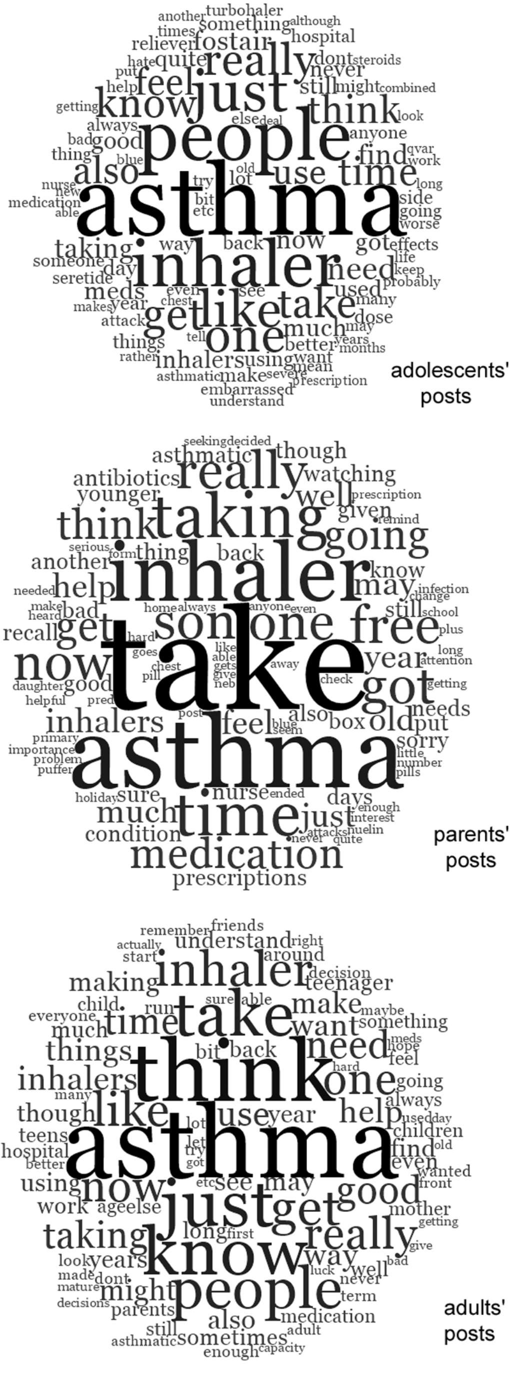

turbohaler
another
something
although
times
hospital
reliever
fostair
hate
quite
really
dont
steroids
put
never
help
feel
just
still
might
combined
getting
know
think
look
always
else
deal
anyone
bad
good
people
thing
blue
find
qvar
work
also
old
try
lot
use
time
long
nurse
new
medication
able
bit
etc
asthma
side
going
worse
taking
way
back
now
got
effects
life
someone
inhaler
day
need
keep
seretide
probably
meds
even
chest
see
used
many
makes
year
take
dose
attack
get
like
much
may
things
one
tell
better
years
months
rather
inhalers
using
want
mean
asthmatic
make
severe
prescription
embarrassed
understand
adolescents' posts
seeking
decided
asthmatic
though
really
antibiotics
watching
younger
well
prescription
think
taking
given
remind
going
serious
form
thing
another
back
know
inhaler
needed
help
may
infection
make
change
heard
bad
home
always
anyone
even
still
school
recall
get
son
one
free
plus
hard
now
like
goes
able
long
chest
gets
away
year
attention
pill
give
neb
check
daughter
good
take
got
getting
helpful
pred
needs
post
inhalers
feel
also
box
old
put
primary
blue
seem
sorry
importance
asthma
little
problem
number
puffer
pills
holiday
sure
nurse
ended
days
much
time
just
enough
interest
condition
attacks
nuelin
never
quite
medication
prescriptions
parents' posts
remember
friends
actually
understand
right
start
around
inhaler
making
decision
teenager
sure
able
child
run
make
everyone
take
maybe
much
time
want
something
meds
things
bit
back
need
hope
feel
think
hard
inhalers
one
going
many
always
though
like
use
year
help
used
day
teens
lot
children
hospital
let
try
asthma
find
old
better
got
even
wanted
using
etc
see
may
front
now
good
work
age
else
just
get
mother
getting
taking
long
first
really
give
look
years
know
way
bad
made
luck
well
mature
dont
might
never
people
term
decisions
parents
also
medication
still
adult
asthmatic
sometimes
capacity
enough
adults' posts
